# Out-of-Pocket Expenditure Among Patients With Diabetes and Hypertension at a Public Secondary Healthcare Facility in Delhi

**DOI:** 10.7759/cureus.85158

**Published:** 2025-05-31

**Authors:** Anita Khokhar, Sunanda Gupta, Pankaj C Nathe, Geeta Yadav, Sanjeev Kumar

**Affiliations:** 1 Community Medicine, Vardhman Mahavir Medical College and Safdarjung Hospital, Delhi, IND

**Keywords:** affordable healthcare, catastrophic health expenditure, cost of healthcare, direct cost, expenses, hypertension, indirect cost, non-communicable disease, out-of-pocket-expenditure, types 2 diabetes

## Abstract

Introduction: Despite national-level improvements in government health expenditure, a significant proportion of patients with chronic conditions like diabetes and hypertension still incur out-of-pocket healthcare expenses, with some facing catastrophic financial burdens. Out-of-pocket expenditure (OOPE) is the direct payments made by individuals for healthcare services at the point of use, excluding any reimbursements from insurance or government schemes. Healthcare spending that exceeds a predefined threshold of a household's income or consumption, potentially pushing families into financial hardship, is referred to as catastrophic health expenditure (CHE). Understanding the extent of OOPE and its potential to cause catastrophic health expenditure is crucial for designing effective financial risk protection measures. This study aims to assess OOPE among persons with diabetes and hypertension visiting a public secondary-level healthcare facility in Delhi.

Methods: A cross-sectional study was conducted among 390 participants using a self-developed questionnaire to collect data on healthcare expenses. The data was collected over a period of six months, from February 2024 to July 2024, with the help of Google Forms (Google Inc., Mountain View, CA, USA). A self-designed, pre-tested questionnaire was developed in English, translated to Hindi, and back-translated to English. The face validity of the questionnaire was assessed by three subject experts. Data was collected by resident doctors. Data was analysed in STATA 14.0 (StataCorp., College Station, TX, USA).

Results: The median age of participants was 58 years (49-64), with 235 (60.3%) being women. The median monthly family income was reported to be 20000 (12000-30000) Indian rupees (INR). The mean monthly OOPE was 174.9 INR, ranging from 0 to 4350 INR. OOPE was incurred by 205 (52.56%) participants. Mean direct expenditure was reported as 156.8 INR (95% CI: 120.8-192.9). Mean indirect expenditure was found to be 22.2 INR (95% CI: 12.1-32.2). Among them, 20 (5.13%) participants had faced CHE.

Conclusion: The findings of this study highlight the heterogeneous nature of OOPE among persons with diabetes and hypertension seeking care at the Rural Health Training Centre in Najafgarh, Delhi. Although many participants reported no OOPE, others experienced significant financial burden across various health service components, including medications, diagnostics, consultations, and hospitalizations. A total of 20 participants experienced CHE. This highlights the need for further research on OOPE determinants and the impact of government interventions.

## Introduction

In India, the proportion of out-of-pocket expenditure (OOPE) out of total health expenditure (THE) has decreased from 64.2% in 2013-14 to 39.4% in 2021-22. At the same time, the government health expenditure (GHE) has increased from 28.6% to 48% of THE. Similarly, social security expenditure on health as percent of THE has changed from 6% to 8.7% [1]. Although the indicators suggest progress, GHE in 2023 was only 2.1% of the total GDP [2]. The impact of OOPE is multifaceted. It not only influences disease control and treatment-seeking behaviours at the individual level and causes impoverishment at the family level, but also has adverse effects at the community level. OOPE was found to have pushed 15% of households below the poverty line. Notably, outpatient care posed a greater burden, with catastrophic health expenditure (CHE) at 47.8% and impoverishment at 15.0%, compared to hospitalization with a CHE of 43.1% and an impoverishment rate of 10.7% [3].

Diabetes and hypertension are chronic diseases that require a lifetime of medication, lifestyle modifications, and regular medical attention for control and prevention of complications. Hence, the affordability of treatment is a significant factor in the hypertension and diabetes care cascade [4]. Reducing out-of-pocket expenses for outpatient care in people with diabetes encouraged more rational health-seeking behaviour, leading to improved overall health [5]. As the burden of non-communicable diseases rises in India, ensuring continuous care for diabetes and hypertension is a critical public health priority [6]. Hence, exploring OOPE among patients of diabetes and hypertension becomes crucial. We noted that though some studies are available about OOPE among persons with diabetes and hypertension at the tertiary healthcare level, there is a dearth of such literature at primary and secondary level healthcare facilities in India.

The objective of the current study is to assess OOPE among persons with diabetes and hypertension visiting the non-communicable disease (NCD) clinic at the Rural Health Training Centre (RHTC) Hospital, Najafgarh, Delhi.

## Materials and methods

A cross-sectional study was carried out at the NCD clinic of RHTC Hospital, Najafgarh. Located in the Southwest district of Delhi, under the Ministry of Health and Family Welfare, RHTC Najafgarh provides clinical services to a population of almost 500,000. All persons with diabetes and hypertension who visited the clinic were included as participants in the study. Pregnant women were excluded. The data was collected over a period of six months, from February 2024 to July 2024, with the help of Google Forms (Google Inc., Mountain View, CA, USA). A self-designed, pre-tested questionnaire was developed in English, translated to Hindi, and back-translated to English. The interview schedule is shown in the appendix. The face validity of the questionnaire was assessed by three subject experts. To minimize recall bias, patients were requested to bring relevant documents (receipts for medicines, investigations, and instruments bought) and were asked to record costs for cross-checking in subsequent visits. All participants were advised about the study in their own language. Informed consent was taken from all participants. Data was collected by resident doctors. Data was analyzed in STATA 14.0 (StataCorp., College Station, TX, USA). Categorical variables were expressed as proportions; in the case of continuous variables, means or medians were calculated as found appropriate. The ethics approval for the study was taken from the institutional ethics committee of Vardhman Mahavir Medical College and Safdarjung Hospital, New Delhi (IEC/VMMC/SJH/Project/2023-12/423, dated 9/2/2024).

The sample size was calculated by an online calculator [7]. Assuming the expected standard deviation of OOPE to be 6452.37 Indian rupees (INR) [8], and margin of error (MOE)=10% of SD, to estimate sample size, the study would require a sample size of 387. Rounding off, the final sample size was taken as 390.

Any payments made by an individual at the point of receiving healthcare services or goods were considered as out-of-pocket expenditure (including reimbursement by insurance company) [9]. We aimed to capture all out-of-pocket expenses incurred by patients at the time of data collection. Given that insurance reimbursements vary in duration and amount, with the possibility of full, partial, or no reimbursement, these uncertainties could not be definitively accounted for at the time of data capture. Therefore, we included all such expenditures in our definition to ensure comprehensive documentation of financial burden. Any person with fasting blood glucose: ≥ 126 milligrams/ deciliters (mg/dl), or two-hour post meal blood glucose: ≥ 200 mg/dl, hemoglobin A1C (HbA1C): ≥6.5%, or random plasma glucose: 200 mg/dl (with symptoms of diabetes), or patients with known diabetes, or patients on medication for diabetes, were enrolled as patient of diabetes. Any patient with blood pressure ≥140/90 mm of Hg (during two or more properly measured blood pressure readings in sitting position), or patients with known hypertension, or patients on medication for hypertension, were enrolled as patients with hypertension [10]. The investigation reports of the latest visit were recorded. Investigation reports that were more than three months old were not recorded. In those cases, the investigations were reopened.

## Results

The median age of the participants was 58 (49-64) years. Almost two-thirds of them were women. The majority, 375 (96.1%), were Hindus. Most of them, 320 (82.1%), had their own house and were married, 327 (83.8%). One-fifth of participants were illiterate, 65 (16.7%) were just literate, and 246 (63.1%) had institutional education. Half of the participants were homemakers, and 32 (8.2%) were retired. Median monthly family income was 20000 INR (12000-30000). While 34 (8.7%) were currently smoking, 29 (7.4%) were former smokers, 27 (6.9%) were currently engaged in chewing tobacco, and 17 (4.4%) were formerly practising it. Alcoholism was prevalent in 25 (6.4%) of participants, and 24 (6.2%) participants had quit alcohol. One-fourth of participants were dyslipidaemic, and nine (2.3%) had a deranged kidney function report. The median body mass index was estimated to be 26 kilograms/metre square (kg/m2) (23.6 - 28.7), in the obesity category. Median random blood glucose was 189 milligrams per decilitre (mg/dl) (139-237). Median fasting blood glucose was 154 mg/dl (117-196). Median systolic blood pressure was found to be 134 millimeters of mercury (mmHg) (125-144), while median diastolic blood pressure was 79.5 mmHg (74-85). The socio-demographic and clinical profile of the participants is described in Table 1.

**Table 1:** Distribution of socio-demographic and clinical variables of the participants (N=390) n=absolute number, 95% CI=95% Confidence Interval, INR: Indian rupee, RBS: random blood sugar, FBS: fasting blood sugar, PPBS: post-prandial blood sugar, HbA1c: glycated haemoglobin, KFT: kidney function test, SBP: systolic blood pressure, DBP: diastolic blood pressure, BMI: body mass index, AYUSH: Ayurveda, Yoga and Naturopathy, Unani, Siddha and Homeopathy.

Variable	n(%)/ median (IQR)
Age (in years)	58 (49-64)
Women	235 (60.3)
Hinduism	375 (96.1)
Migrant worker	46 (11.7)
Own house ownership	320 (82.1)
Married	327 (83.8)
Education	
Illiterate	79 (20.2)
Just literate	65 (16.7)
Primary school	31 (7.9)
Middle school	86 (22.1)
High school	62 (15.9)
Senior secondary	30 (7.7)
Graduate	30 (7.7)
Post graduate	7 (1.8)
Occupation	
Homemaker	205 (52.6)
Skilled manual labour	61 (15.6)
Self-employed and small	35 (8.9)
Retired	32 (8.2)
Sales and marketing executive	19 (4.9)
Clerical	10 (2.6)
Others	28 (7.2)
Monthly family income (INR)	20000 (12000-30000)
Number of family members	5 (3-6)
Smoking status	
Current	34 (8.7)
Former	29 (7.4)
Never	327 (83.8)
Tobacco chewing status	
Current	27 (6.9)
Former	17 (4.4)
Never	346 (88.7)
Alcohol user	
Current	25 (6.4)
Former	24 (6.2)
Never	341 (87.4)
RBS (mg/dl)	189 (139-237)
FBS (mg/dl)	154 (117-196)
PPBS (mg/dl)	242 (189.5-299)
HbA1c (%)	8.2 (6.8-10.2)
Dyslipidaemia	
Yes	97 (24.9)
No	150 (38.5)
No reports available	143 (36.7)
KFT	
Within normal limits	264 (67.7)
Deranged	9 (2.3)
No reports available	117 (30.0)
SBP (mm Hg)	134 (125-144)
DBP (mm Hg)	79.5 (74-85)
BMI (kg/m^2^)	26 (23.6 – 28.7)
Diagnosis	
Only Diabetes	167 (42.8)
Only Hypertension	65 (16.7)
Diabetes and hypertension	158 (40.5)
Duration of diabetes (in months)	60 (24 -108)
Duration of hypertension (in months)	36 (12-72)
Treatment	
Allopathy only	375 (96.2)
Allopathy and AYUSH	15 (3.8)

The majority, i.e, 375 (96.2%), were seeking treatment from Allopathy practitioners, while the rest were taking both Allopathy and Ayurveda, Yoga and Naturopathy, Unani, Siddha and Homeopathy (AYUSH) treatment. Among total participants, 158 (40.5%) were both diabetic and hypertensive, 167 (42.8%) were diabetic, and 65 (16.7%) were only hypertensive. Median duration of diabetes among participants was 60 months (24-108), and that of hypertension was 36 months (12-72).

Only 13 (3.3%) participants received reimbursement for their medical expenses. Two participants received reimbursement from the government (as a job facility), while the rest had private insurance. Since the distributions were found to be non-parametric, median and interquartile range (IQR) were reported.

Table 2 describes out-of-pocket expenditure incurred among study participants. Mean expenditure of patients per monthly outdoor patient department (OPD) visit was found to be 174.9 INR (95% CI:136.8-213.1), while it ranged between 0 to 4350 INR. This included the cost of medication, travel from home to RHTC, wage loss of patient and attendant, cost of exercise, cost of lifestyle modification, and consumables for monitoring devices like cotton, spirit, needle, and glucometer strips. The mean of one-time expenditure of patients was noted as 578 INR (95% CI: 493.4- 662.4). This included the cost of monitoring devices, i.e., sphygmomanometer and/or glucometer. Hospitalization cost was incurred by 38 participants. The median expenditure was found to be zero in all categories of expenditure. The median and IQR for the monthly expenditure of patients was found to be 0 INR (0-240), and the one-time expenditure of patients was reported to be 0 INR (0-1000). Though at RHTC Najafgarh, consultation, medication, and investigations are free of cost from the patients’ side, the patients incurred OOPE when they opted for a second opinion and investigations from private resources, and for medication in case of stockout in hospital supplies. About 20% of the total participants could provide us with receipts. Another 10% of the population maintained a record (self-recorded) of the cost prospectively. The same was true for indoor services at private hospitals.

**Table 2:** Out-of-pocket expenditure (OOPE) among participants (N=390) INR: Indian rupee, Min: minimum, Max: maximum, 95% CI: 95% confidence interval.

OOPE reasons	Range (Min-Max) (in INR)	Mean (95% CI) (in INR)
Expenditure of patients per monthly OPD visit	0-4350	174.9 (136.8-213.1)
One-time expenditure of patients	0-3500	578 (493.4- 662.4)
Total consultation cost over past one year	0-100000	538.1 (18-1058.1)
Investigations cost over last one year	0- 6000	49.5 (13.4- 85.6)
Hospitalization cost over last one year	0-500000	28077.6 (207.4- 55947.9)

While direct expenditure included expenses for medicine, dietary modification, exercise, travelling, investigations, and consumables for monitoring devices, indirect expenditure included wage loss and food expenses. Mean direct expenditure was reported as 156.8 INR (95% CI: 120.8-192.9). Mean indirect expenditure was found to be 22.2 INR (95% CI: 12.1-32.2). Table 3 describes the direct and indirect expenditure.

**Table 3:** Direct and indirect monthly expenditure among participants (N=390) INR: Indian rupee, Min: minimum, Max: maximum, 95% CI: 95% confidence interval.

	Monthly expenditure (in INR) Range (Min-Max)	Monthly expenditure (in INR) Mean (95% CI)
Direct expenditure	0-4350	156.8 (120.8-192.9)
Indirect expenditure	0-800	22.2 (12.1-32.2)

Among all participants, 205 (52.6%) incurred OOPE over the past year. CHE was calculated as expenditure on health being more than 10% of total household income [11]. CHE was noted in 20 (5.13%) participants.

## Discussion

An increase in government spending on healthcare plays a significant role in reducing the financial burdens faced by households. Between 2014-15 and 2021-22, the GHE as a share of the country's THE increased from 29% to 48%. During this period, OOPE as a share of THE dropped from 62.6% to 39.4% [12]. In this backdrop, our study adds an essential source of evidence about the out-of-pocket spending on diabetes and hypertension. While 185 (47.4%) participants incurred no healthcare costs for these diseases, the remaining participants had to bear some expenses. Of the 20 patients (5.1%) who incurred CHE, 14 were from the lowest income quintile. Bivariate analysis revealed a significant association between income quintiles and CHE (p < 0.001). The median monthly family income among patients having CHE was calculated to be 4250 INR (IQR: 1000-13,500). These findings highlight a higher risk of CHE among patients from families earning less. We see a broad variation in values across each category, it suggests that healthcare expenses cover a very wide range. Therefore, the risk of impoverishment remains, despite the overall improvement in national indicators.

The RHTC, Najafgarh, as a government institute, offers free consultations, medications, and diagnostic services. However, medication stockouts occasionally necessitate out-of-pocket purchases by patients. Some patients opt for external diagnostic services for reasons such as reduced waiting times or shorter fasting requirements. Additionally, second opinions from private practitioners are sought, often driven by factors like evening availability and shorter consultation waits.

In a study carried out in north India, the average OOPE for a diabetes patient visiting a tertiary care facility was 4,381 INR (95% CI: 3,786-4,976). In the case of hypertension, the mean OOPE for outpatient consultation was 1,427 INR (95% CI: 1,278-1,576). In our study, the reported OOPE was much lower. This can be attributed to the fact that our clinic was not at a tertiary care facility and primarily catered to patients with fewer complications or less advanced medical needs. Most patients came from nearby areas, resulting in shorter travel expenses and no lodging expenses. Also, RHTC does not have indoor healthcare facilities and specialist/superspecialist services yet; hence, patients visit mostly for regular checkups. The lower patient load also meant shorter waiting times, which minimized wage loss and other indirect expenses. Five percent of our patients had incurred CHE; this proportion was, however, similar to available literature [13]. While we did not find any associated factors with CHE in our study, this study reported female sex, disease with complications, and the poorest quintile to be associated with CHE [13]. A study from West Bengal, with hypertensive participants, revealed that the total cost of seeking complete care was 306.49 INR (95% CI: 257.65-355.33). Only 20% of the participants sought treatment from a public facility in this study, whereas our study completed the enrollment of patients from a public facility. Longer duration of disease, health seeking from non-public organizations, presence of co-morbidities, and poorer socioeconomic groups were found to be associated with higher OOPE in this study [14]. In a study from Punjab, the median direct annual expenditure of diabetes was reported to be 34100 INR (18000-56300), while the indirect annual expenditure was found to be 4200 INR (2400-7200) [15]. In a study from Rajasthan, the total mean expenditure for the past three months for diabetes or hypertension patients was estimated to be 9014.37 INR (SD: ±6452.37). The mean outpatient expense for the last three months was noted to be 3518.30 INR (SD: ±2133.05), and for inpatient care, it was 7405.18 INR (SD: ±5152.86) [8]. Another study from a tertiary care hospital in Uttarakhand reported that the mean annual direct cost among diabetics was estimated to be 7338.9 INR [16]. The costs are reported to be higher than our study findings, primarily because of the different healthcare levels of the study settings. In a meta-analysis of Indian studies, the mean expenditure on diabetes and its complications was calculated as 15,535 INR per year. On average, the OPD charges were found to be 3%-5% of the total annual income of the individual. In case of complications and hospitalization, the average expenditure was higher (21%), which averages to around 11,000 INR [17]. A study from a South Indian secondary healthcare centre, the median expenditure of diabetic patients in one year was found to be 246 INR (Range: 3-24,000) [18]. A study from Mysuru reported mean total OOPE was 1173.06 (INR), with a SD of 1424.54 (INR), and the mean direct cost incurred was 1141.03 [19]. Overall, huge variations in OOPE for NCD are reported across different states of India [20].

Strengths and limitations

We made efforts to triangulate the data by comparing patient self-reported amounts with available billing records, in order to verify the collected information, wherever feasible. However, we could not achieve this in all cases. Hence, recall bias can be present. The study could not account for certain health system factors like the availability of medicines, diagnostic investigations, and cross-referrals, as stockout patterns were unpredictable and irregular. This may have influenced out-of-pocket expenditure. We sought expense information from attendants when the patient appeared to be unreliable. This approach was particularly effective in cases involving elderly or dependent patients accompanied by their caregivers. Given that the clinic has a fixed cohort of patients who visit repeatedly, we frequently had multiple opportunities to fill in missing data and cross-check it with available patient records. This ultimately reduced the amount of missing data. Data collection was conducted by only three residents, all of whom underwent standardized, consensus-based training. As a result, the collected data exhibited minimal inter-interviewer variations. On multiple occasions, patients rounded off values to the nearest whole number, often ending in zeros, which provided us with approximate rather than precise amounts. A key limitation of the study is its cross-sectional design, which involves data collection at a single point in time and also restricted the ability to establish causal relationships. Furthermore, as this was an institution-based study conducted at a single center in Delhi, the generalizability of the findings is limited.

## Conclusions

In conclusion, the study highlights the variability in OOPE among persons with diabetes and hypertension visiting the Rural Health Training Centre in Najafgarh, Delhi. While a significant number of participants incurred no OOPE, others faced wide-ranging costs, for medication, investigations, consultation charges, hospitalization, and others. CHE was noted in 20 participants. Despite the improvements in national health indicators, the risk of financial hardship due to healthcare expenses persists. Future research should also focus on exploring the determinants of OOPE and assessing the long-term effects of government interventions aimed at reducing these expenses.

Medication stockouts at government healthcare facilities drive OOPE among patients, underscoring the need for immediate action. A robust and timely drug procurement system is crucial to accurately track consumption and ensure an uninterrupted supply of essential medicines. It is important to explore the underlying factors that lead patients to seek diagnostics and consultations from private providers despite free services being available in the public sector. Probable factors such as waiting times and extended evening availability should be considered at government facilities. Comprehensive patient counselling is essential to discourage unnecessary second opinions. A strong referral system connecting to higher government facilities will also be helpful. Additionally, further research at primary and secondary care levels is needed to understand patients' financial constraints, as their healthcare needs differ from those in tertiary care. Future studies should also investigate the drivers of OOPE and assess the long-term effectiveness of government initiatives aimed at mitigating these costs.

## Appendices

**Table 4:** Interview schedule used for data collection

Socio-demographic details
PID:
Name:
Interviewer’s name:
Date:
Phone number:
Age (in years):
Sex: male/female/other
Religion: Hinduism/Islam/Christianity/other
Marital status: Married/unmarried/ divorced/separated/widowed/living in
Education: Illiterate/ Just Literate/ Primary school/ Middle school/ High school/ Higher secondary/ Graduate/ Post graduate
Which of the following best describes your main work status/ occupation over the past 12 months? Professional/Medium or large/ Middle / Senior Executive/officer in organization/ Agricultural land owner/ Sales and Marketing executives/ Clerical/ Self-employed and small/ Skilled manual laborer/ Unskilled manual or agricultural laborer/ Student/ Homemaker/ retired/ unemployed (able to work)/ unemployed (unable to do any work)
Smoker: Never/ Former/ Current
If current/ former user: Type of product used: Cigarette/ Bidi/ Cigar/ Hookah/ Other Mention if other:
Numbers used per day:
Duration of use (in years):
Family income per month (average of last one year):
Household expenditure per month (average of last one year):
Alcohol use: Never/ Former/ Current
If current/ former user: Type of product used: Beer/Rum/Whisky/Vodka/Wine/Other Mention if other:
Percentage of alcohol in the used drink:
Amount used per day (ml):
Duration of use (in years):
Smokeless tobacco use: Never/ Former/ Current
If current/ former user: Type of product used: Gutkha/ Khaini/ Zarda/ Pan Masala/ other
Mention if other: Name of the brand:
Amount used per day (mg):
Duration of use (in years):
RBS:
FBS:
PPBS:
HbA1c:
Dyslipidaemic: Yes/No
KFT deranged: Yes/No
Blood pressure reading 1:
Blood pressure reading 2:
Any other co-morbidity:
Height:
Weight:
Diagnosis: Diabetes/ Hypertension/Diabetes &Hypertension
1^st^ visit to this OPD: Yes/No
Duration of DM since diagnosis (in months):
Duration of HTN since treatment (in months):
Treatment: Allopathy/Homeopathy/Ayurveda/ Naturopathy and Yoga/Unani/Siddha/Mixopathy
Out-of-pocket expenditure: (Mention cost for each)
Monitoring device:
Glucometer
Sphygmomanometer:
Monitoring tools: (in past one month)
Glucometer strips:
Needles:
Cotton:
Spirit:
Details of medicines bought in past one month:(include supplements and AYUSH medications)
Sr. No. Name of medicine Dose Frequency per day
1
2
3
4
5
6
7
8
Details of medicines bought in past one month: (include supplements)
Sr. No. Name of medicine Dose Frequency per day
1
2
3
4
5
6
7
8
Details of investigations done in past one year:
Sr. No. Name of investigation Centre of investigation (Government/private) Total cost in past one year
1
2
3
4
5
6
7
8
Consultation charges in past one year:
Sr. No. Allopathy/Homeopathy/Ayurveda/ Naturopathy and Yoga/Unani/Siddha/Undetermined Centre of consultation (Government/private) Cost per visit
1
2
3
4
5
6
7
8
Tips for getting services:
Sr. No. Cost per visit
1
2
3
4
5
6
7
8
Cost of Exercise for past one year
Sr. no. Type of exercise Duration (in months) Cost per month Total cost
1
2
3
4
Cost for any dietary modification done specifically for your disease since past one year:
Sr. No. Name of item Duration (in months) Cost per month
1
2
3
4
Cost for travelling to healthcare facility in past one year:
Sr. No. Cost per visit
1
2
3
4
5
6
7
8
9
10
Wage loss of patient per healthcare facility visit:
Wage loss of attendant per healthcare facility visit:
Cost of food for patient per healthcare facility visit:
Cost of food for attendant per healthcare facility visit:
Any other significant healthcare related cost in past one year:
Sr. No. Reason Total cost
1
2
Cost for hospitalization in past one year:
Sr. No. Reason for hospitalization Total cost
1
2
Any reimbursement: (encircle) Yes/ No. If yes,
Name Govt./Private/ Employment-related Full/partial reimbursement Amount Monthly premium Duration of premium paying till date (in months)
1
2
Any medical facility entitlement: Yes/ No. If yes, mention:

## References

[1] (2024). National health accounts. https://nhsrcindia.org/national-health-accounts-records.

[2] (2024). Economic survey. https://www.indiabudget.gov.in/economicsurvey/.

[3] Nanda M, Sharma R (2023). A comprehensive examination of the economic impact of out-of-pocket health expenditures in India. Health Policy Plan.

[4] Wang J, Tan F, Wang Z (2024). Understanding gaps in the hypertension and diabetes care cascade: systematic scoping review. JMIR Public Health Surveill.

[5] Du W, Liu P, Xu W (2022). Effects of decreasing the out-of-pocket expenses for outpatient care on health-seeking behaviors, health outcomes and medical expenses of people with diabetes: evidence from China. Int J Equity Health.

[6] Arokiasamy P (2018). India’s escalating burden of non-communicable diseases. Lancet Glob Health.

[7] (2024). Sample size calculator. https://www.statskingdom.com/50_ci_sample_size.html..

[8] Mehta R, Mantri N, Goel AD, Gupta MK, Joshi NK, Bhardwaj P (2022). Out-of-pocket spending on hypertension and diabetes among patients reporting in a health-care teaching institute of the Western Rajasthan. J Family Med Prim Care.

[9] (2024). Health care financing indicators. https://nhsrcindia.org/hcf-INDICATORS.

[10] (2025). Training module for medical officers for prevention, control and population level screening of hypertension, diabetes and common cancer (oral, breast & cervical). https://mohfw.gov.in/sites/default/files/Training%20Module%20for%20Medical%20Officers%20for%20Prevention%2C%20Control%20and%20Population%20Level%20Screening%20of%20NCDs_1.pdf.

[11] (2024). Catastrophic health spending (and related indicators). https://www.who.int/data/gho/data/themes/topics/financial-protection/GHO/financial-protection.

[12] (2024). Union Health Ministry releases National Health Accounts Estimates for India 2020-21 and 2021-22. Estimates for India.

[13] Brar S, Kaur G, Muniyandi M (2023). Cost of screening, out-of-pocket expenditure & quality of life for diabetes & hypertension in India. Indian J Med Res.

[14] Chakraborty S, Rai RK, Biswas AK, Barik A, Gurung P, Praveen D (2022). Health care seeking behaviour and financial protection of patients with hypertension: a cross-sectional study in rural West Bengal, India. PLoS One.

[15] Kansra P, Oberoi S (2023). Cost of diabetes and its complications: results from a STEPS survey in Punjab, India. Glob Health Res Policy.

[16] Nath B, Gupta SD, Kumari R (2020). Effect of comorbidities on direct cost among type 2 diabetes mellitus (T2DM) patients in tertiary care government hospital in Uttarakhand, India: a primary data analysis of out of pocket expenditure. Diabetes Metab Syndr.

[17] Sathyanath S, Kundapur R, Deepthi R, Poojary SN, Rai S, Modi B, Saxena D (2022). An economic evaluation of diabetes mellitus in India: a systematic review. Diabetes Metab Syndr.

[18] Shobhana R, Rao PR, Lavanya A, Williams R, Vijay V, Ramachandran A (2000). Expenditure on health care incurred by diabetic subjects in a developing country — a study from southern India. Diabetes Res Clin Pract.

[19] Choedon FNU, Murthy MR, Shree A, N C (2023). Assessment of out-of-pocket expenditure on noncommunicable diseases in urban slum of Mysuru city. Indian J Med Spec.

[20] Menon GR, Yadav J, John D (2022). Burden of non-communicable diseases and its associated economic costs in India. Soc Sci Humanit Open.

